# Optimizing dwell time weight for interstitial needles in intracavitary/interstitial hybrid brachytherapy: balancing tumor coverage with organ sparing using the inverse planning technique

**DOI:** 10.1093/jrr/rraf025

**Published:** 2025-05-14

**Authors:** Jun Takatsu, Naoya Murakami, Noriyuki Okonogi, Tatsuya Inoue, Kotaro Iijima, Yoichi Muramoto, Yasuo Kosugi, Terufumi Kawamoto, Tatsuki Karino, Yasuhisa Terao, Naoto Shikama

**Affiliations:** Department of Radiation Oncology, Juntendo University Graduate School of Medicine, 2-1-1 Hongo, Bunkyo-ku, Tokyo 113-8421, Japan; Department of Radiation Oncology, Juntendo University Graduate School of Medicine, 2-1-1 Hongo, Bunkyo-ku, Tokyo 113-8421, Japan; Department of Radiation Oncology, Juntendo University Graduate School of Medicine, 2-1-1 Hongo, Bunkyo-ku, Tokyo 113-8421, Japan; Department of Radiation Oncology, Juntendo University Graduate School of Medicine, 2-1-1 Hongo, Bunkyo-ku, Tokyo 113-8421, Japan; Department of Radiology, Juntendo University Urayasu Hospital, 2-1-1 Tomioka Urayasu-shi, Chiba 279-0021, Japan; Department of Radiation Oncology, Juntendo University Graduate School of Medicine, 2-1-1 Hongo, Bunkyo-ku, Tokyo 113-8421, Japan; Department of Radiation Oncology, Juntendo University Graduate School of Medicine, 2-1-1 Hongo, Bunkyo-ku, Tokyo 113-8421, Japan; Department of Radiation Oncology, Juntendo University Graduate School of Medicine, 2-1-1 Hongo, Bunkyo-ku, Tokyo 113-8421, Japan; Department of Radiation Oncology, Juntendo University Graduate School of Medicine, 2-1-1 Hongo, Bunkyo-ku, Tokyo 113-8421, Japan; Department of Radiation Oncology, Juntendo University Graduate School of Medicine, 2-1-1 Hongo, Bunkyo-ku, Tokyo 113-8421, Japan; Department of Obstetrics and Gynecology, Faculty of Medicine, Juntendo University, 2-1-1 Hongo, Bunkyo-ku, Tokyo 113-8421, Japan; Department of Radiation Oncology, Juntendo University Graduate School of Medicine, 2-1-1 Hongo, Bunkyo-ku, Tokyo 113-8421, Japan

**Keywords:** intracavitary/interstitial hybrid brachytherapy, HIPO, cervical cancer, IGABT, interstitial needle dwell weight

## Abstract

The recommended dwell time weight of the needle in intracavitary/interstitial hybrid brachytherapy (HBT) has been 10–20%. This study aimed to investigate the correlation between the weight constraint of the needle and normal organ doses in uterine cervical cancer HBT. This study included 30 cervical cancer patients who received HBT with tandem/ovoid applicators. In our clinical practice, treatment plans were generated without the constraint of the dwell time weight of the needle. The cases where this weight exceeded 20% were replanned. An inverse planning technique with locking downscaled needle dwell time was used to reproduce isodose lines of clinical plans. Replanning repeated with downscaling of the dwell time until the weight of the needle fell <20% (Needle-Lock plan). The Needle-Lock plans were rescaled to coincide with the high-risk clinical target volumes D_90_ of clinical plans. D_2cc_ in normal organs and the overdose area >200% of the prescribed dose were evaluated. In 17 of 30 (56.7%) clinical plans, the weight of the needle exceeded 20%. The rectum, bladder and sigmoid colon D_2cc_ significantly increased with the Needle-Lock plan. The overdosage area also increased significantly (*P* < 0.01). The correlations between the needle number and the increase of D_2cc_ in the rectum and sigmoid colon (*P* < 0.01) were statistically significant. Limiting needle dwell time weight by 10–20% increased bladder and rectum doses, especially with multiple needles. These findings suggest that needle dwell time weight recommendations could need to be reconsidered based on individual and institutional situation.

## INTRODUCTION

Brachytherapy for uterine cervical cancer has transitioned from 2D point A prescription to 3D image-guided adaptive brachytherapy (IGABT) [1, 2]. The intErnational study on MRI-guided BRachytherapy in locally Advanced CErvical cancer (EMBRACE) study demonstrated the optimal prescription target dose and its constraints for normal tissues with magnetic resonance (MR)–based IGABT in cervical cancer [3, 4]. Brachytherapy can be safely performed by assessing the D_90_ of high-risk clinical target volumes (CTV_HR_) and the D_2cc_ of organs at risk (OARs) using computed tomography (CT) or MR images. Moreover, prescription doses to bulky or irregularly shaped tumors can be delivered using hybrid brachytherapy (HBT), combining intracavitary and interstitial techniques [5–8].

In the treatment planning process for HBT, the dwell time weight of the additional needles should not increase relative to that of the intracavitary applicators. Previous reports recommended that the dwell time weight of needles should be limited to 10–20% of that of the tandem [9–13]. This constraint prevents the occurrence of high-dose areas in parts of the CTV_HR_ outside the uterus to avoid unexpected late radiation-related toxicities. However, in clinical practice, there are cases in which needle weights are relatively higher than the previously recommended constraint, especially for irregularly shaped tumors. Furthermore, various applicators have been developed [14] that can enable the flexibility of the dwell time weight constraint of the needles on a case-by-case basis.

The quality of treatment plans for HBT with the forward planning approach depends heavily on the experience of each planner. Sometimes, dose delivery to the CTV_HR_ is prioritized over the dose constraints of OARs. This makes it challenging to ensure reproducibility of HBT using the forward planning approach. Then, we focused on the inverse planning approach, commonly used in external beam radiotherapy (EBRT). It has recently been implemented in HBT treatment planning [10–15]. Introducing inverse planning potentially reduces inter-planner variation and enables reproducible treatment planning.

This study aims to investigate the correlation between the dwell time weight of needles relative to intracavitary applicator dwell time weight and OAR doses in uterine cervical cancer HBT using inverse planning, focusing on the recommended constraint of 10–20%. In our department, we do not strictly adhere to the recommendation of the 10–20% rule, where a better dose distribution is achieved with a needle weight exceeding 10–20%. This study focuses on such cases with their needle weights exceeding 10–20% and assesses if it is necessary to adhere to the 10–20% rule or not. When the correlation between the dwell time weight of needles and the high-dose area exceeded 200% of the prescribed dose, the hyper-dose sleeve was also evaluated.

## METHOD

### Brachytherapy treatment

Thirty locally advanced cervical cancer patients who underwent CT-based HBT between January 2023 and July 2024 were selected. All patients receiving HBT with tandem/ovoid as the intracavity applicator during this study period were included. Table 1 shows the detailed patient characteristics. According to the eighth edition of the International Union against Cancer/American Joint Committee on Cancer (UICC/AJCC) staging system, the T stage was classified. This study was approved by the ethics committee of our institution and was conducted according to the Declaration of Helsinki. Informed consent was obtained from each patient using the ‘opt-out’ method.

**Table 1:** Characteristics of patients

FIGO (2018)	
Stage IIA	2
Stage IIB	11
Stage IIIB	1
Stage IIIC	15
Stage IV	1
T Stage (8th edition of UICC/AJCC)	
T2a2	2
T2b	20
T3b	6
T4a	2
N Stage	
N0	13
N1	14
N2	3
Histology	
Squamous cell carcinoma	26
Adenocarcinoma	3
Adenosquamous cell carcinoma	1
Applicator	
Fletcher CT/MR tandem/ovoid	11
Fletcher-Suit Asian Pacific tandem/ovoid	6
Geneva tandem/ovoid	13

FIGO = International Federation of Gynecology and Obstetrics, UICC/AJCC = International Union against Cancer/American Joint Committee on Cancer.

The tandem/ovoid applicators included Fletcher CT/MR, Fletcher-Suit Asian Pacific or the Geneva (Elekta, Stockholm, Sweden) applicators. All patients were injected with hyaluronic acid gel (MucoUp; Seikagaku Co., Tokyo, Japan) into the rectovaginal and vesicoureteral septum to minimize rectal and bladder doses [16]. One to seven additional interstitial needles were inserted through the perineum or vagina. The median number of needles inserted was two. Planning CT images were acquired in the treatment room using an Aquilion LB system (Canon Medical Systems Corp., Tochigi, Japan). Patients were positioned in lithotomy, and the thickness of the CT slice was 2 mm. CT images were imported into Oncentra version 4.6 (Elekta).

The bladder, rectum, sigmoid colon, small bowel and CTV_HR_ were contoured by several experienced radiation oncologists. CTV_HR_ contouring was performed according to the standards of the Japanese Society for Radiation Oncology [17]. The step size for the dwell position of the activated source was set to 5 mm. HBT was performed after or near the end of EBRT and was delivered over three to four sessions of 6 Gy each, considering the dose constraints of OARs [18]. All patients received EBRT to the whole pelvis without central shielding to 45–50 Gy in 25–28 fractions, with a boost of 6–14 Gy to metastatic lymph nodes for N1 cases [19]. EBRT and brachytherapy doses were converted to biological equivalent doses in 2 Gy (EQD_2_) for dose summarization. In the EQD_2_ conversion process, α/β = 3 Gy for OAR and α/β = 10 Gy for CTV_HR_ were used, respectively. The HBT plans were generated to meet the OAR dose constraints of 80 Gy for the bladder, 65 Gy for the rectum, 65 Gy for the sigmoid colon and 60 Gy for the small intestine at D_2cc_ in dose summation. The HBT plans were also designed to achieve CTV_HR_ D_90_ >85 Gy while meeting the dose constraints of the OARs [3]. The dwell time weight of needles relative to that of the intracavitary applicator was not considered when generating HBT plans that prioritized OAR dose constraints and CTV_HR_ dose delivery.

### Generation of plans for the dwell time weight constraint of needles

**Fig. 1:**
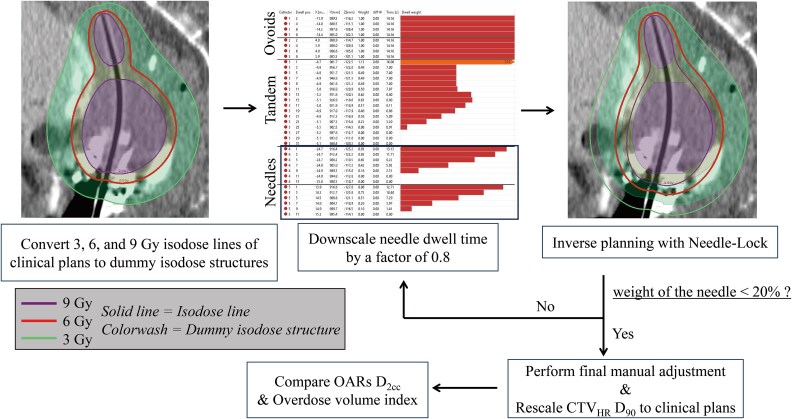
Workflow for plan generation with needle dwell time weight constraints using the inverse planning technique.

**Fig. 2:**
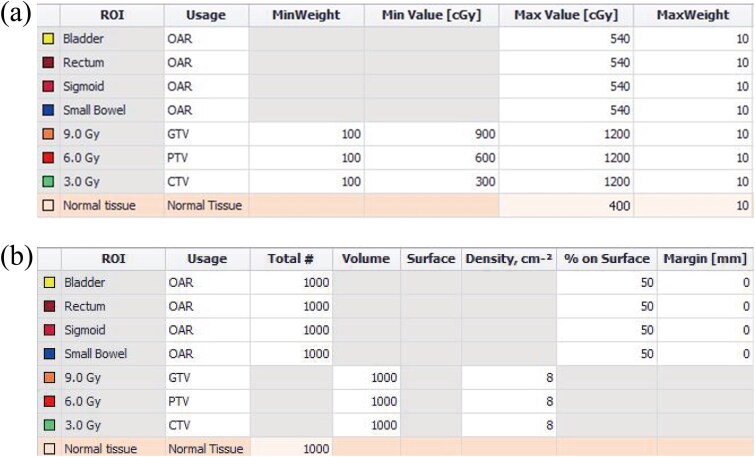
Optimization parameters in the HIPO calculation. (a) Dose volume objectives and (b) sampling point settings for each structure.

The dwell time weights of needles for tandem/ovoid applicators in clinical plans were evaluated using the treatment plan for the first HBT session. Then, the following process was performed for plans with needle weights exceeding 20%. Figure 1 shows the workflow for generating plans that limited the dwell time weight of needles up to 20%. The first step was to convert the 3-, 6- and 9-Gy isodose lines from the clinically generated plans to dummy isodose structures with the MIM software version 6.5.9 (MIM Software Inc., Cleveland, OH). Next, the dwell times of needles were scaled down by a factor of 0.8. To gradually reduce the needle dwell time, the value of 0.8 was chosen empirically. By locking the needle dwell time, the isodose lines at 3, 6 and 9 Gy were optimized to reproduce the dummy isodose structures. Hybrid inverse planning optimization (HIPO) was used for optimization calculations. The optimization algorithm of HIPO has been reported in previous studies [14, 20]. The optimization parameters used in the HIPO calculations are shown in Fig. 2. The dwell time gradient restriction was set to 0.6 [12, 21]. After optimizing, the dwell time weight of needles to tandem/ovoid was recalculated. When this dwell time weight of needles was still >20%, the same process was repeated. Finally, the needle dwell time was manually adjusted to keep it within 20%. To compare the clinical plans with no dwell time weight constraint of needles and retrospectively generated plans with dwell time weight constraint of needles <20% (Needle-Lock plans), the CTV_HR_ D_90_ of Needle-Lock plans was rescaled to be consistent with clinical plans. In this study, in order to provide the same dose as the clinical plan in the regions of the ovoid and tandem tip that are not included in the CTV_HR_ [22], inverse planning was performed on dummy isodose structures, with the needle dwell time constraints, rather than on the CTV_HR_ or OAR.

Clinical plans and Needle-Lock plans were compared for OARs D_2cc_. Correlations between the differences in the OARs D_2cc_ and the number of needles was evaluated. The dose difference of OARs was defined as follows:


$$ Dose\ difference=\frac{OAR\ {D}_{2 cc}^{Needle- Lock}- OAR\ {D}_{2 cc}^{Clinical}}{OAR\ {D}_{2 cc}^{Clinical}}\times 100 $$

where OAR D_2cc_^Needle-Lock^ and OAR D_2cc_^Clinical^ are D_2cc_ values for each OAR for the Needle-Lock and clinical plans, respectively.

In addition, because the original purpose of limiting needle dwell time <20% was to limit the occurrence of high-dose areas, the volume of overdose >200% of the prescription dose, also known as hyper-dose sleeve, was mutually compared between the clinical and the Needle-Lock plans. Thus, the overdose volume index (OI) was calculated [23]. Palled *et al*. evaluated the overdose within the target volume, which resulted in insufficient dosimetric evaluation around the ovoid and tandem tips. Therefore, our clinical protocol delineated only the CTV_HR_ as the target, and the OI was redefined as follows:


$$ OI=\frac{V_{200\%}}{V_{100\%}} $$

where *V*_100%_ and *V*_200%_ are the volumes surrounded by the prescribed dose and those more than twice the prescribed dose on the CT images, respectively.

### Virtual organs at risk simulation

To apply the results of this study to institutions that do not perform hydrogel spacer injections and increase the generalizability of this research, an additional virtual OARs simulation study was conducted.

**Fig. 3:**
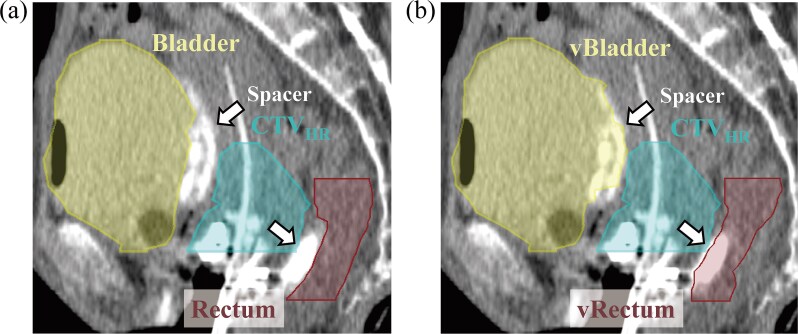
Generation of the virtual OARs. CT images of patient 5: (a) clinical plan and (b) the virtual OARs simulation. The arrow represents the hydrogel spacer.

The first step in this simulation was to generate the virtual OARs that were located in anatomical places if there was no spacer materials. The rectum contoured by the radiation oncologist was shifted to the CTV_HR_ side on axial slices containing hydrogel spacer and smoothly connected to the upper slices without hydrogel spacer. As a result, a virtual rectum was generated when the hydrogel spacer was not inserted. The different approach for the bladder was based on the characteristics of hydrogel spacer used in this study (MucoUp). While MucoUp effectively maintained spacing between the vagina and rectum, it was less effective at creating consistent spacing between the vagina and bladder. This was likely due to MucoUp’s relatively low molecular weight (500–1200 kDa) compared to Suvenyl (1500–3900 kDa) used in previous studies and limited space in the bladder side compared to the rectal side, causing the hydrogel to flow laterally when inserted between the vagina and bladder [16, 24]. Therefore, instead of shifting the bladder position, the hydrogel spacer volume was included in the bladder contour. These virtual OARs were defined as vRectum and vBladder, respectively. This is because the rectum could be kept at a sufficient distance from the CTV_HR_ with the hydrogel spacer, but the bladder side could not be kept at a sufficient distance with the hydrogel spacer. Therefore, virtual OARs were processed in this way. Figure 3 shows the generated virtual OARs.

Next, a single radiation oncologist adjusted the dose distribution of the clinical plan generated with the hydrogel spacer for the virtual OARs. The plan was generated for the CTV_HR_ and virtual OARs using a protocol based on actual clinical practice. The plan manually generated by the radiation oncologist for the virtual OARs was defined as the virtual OARs Manual plan. In this study, virtual OARs Manual plans were retrospectively generated for cases where the needle dwell time weight exceeded 20% in the clinical plan. Since clinical plans included three or four HBT sessions, virtual OARs Manual plans were generated by standardizing the HBT schedule to four sessions. At our institution, four-session schemes of HBT are generally conducted, and the goal CTV_HR_ D_90_ is set to 85 Gy or more by summation with EBRT and brachytherapy doses. However, in cases where the delivered doses to OARs could be sufficiently reduced by inserting the hydrogel spacer, the session number of HBT could be reduced to three by increasing the dose per HBT session [18]. For the virtual OARs simulation, which was designed to evaluate scenarios without the hydrogel spacer and demonstrate the applicability of findings to institutions that do not use spacers, simulation was conducted with the four-session scheme as a more conservative strategy. In the treatment plan using the virtual OARs simulation, the dose distribution was generated so that the summation of EBRT and brachytherapy would exceed 85 Gy for the CTV_HR_ D_90_ in four brachytherapy sessions. At our institution, the dose constraints of each brachytherapy session for the rectum and bladder are set at 448 cGy and 545 cGy, respectively, according to the GEC-ESTRO recommended dose constraints [3]. The radiation oncologist generated the treatment plan with assessing the balance between these dose constraints and the dose delivery to CTV_HR_. For these virtual OARs Manual plans, plans were also generated with the needle dwell time weight reduced to 20%. These plans were defined as virtual OARs Needle-Lock plans.

As with the clinical plans and the Needle-Lock plans, the correlation between the dose difference of the virtual OARs and the number of needles was evaluated in the virtual OARs simulation.

### Statistical analysis

Clinical plans were categorized based on a needle dwell weight of ≥20% and those of ≤20%, with Mann–Whitney *U* tests used to compare CTV_HR_ volumes. Next, Wilcoxon signed-rank test was used to compare the dose volume histogram (DVH) and several plan parameters between clinical and Needle-Lock plans. In addition, statistical correlations between the number of needles and variations in OARs D_2cc_ were evaluated using Spearman’s rank correlation coefficient (*r*_s_). Depending on the absolute value of *r*_s_, the correlation was generally classified as 0.20–0.39, ‘weak’; 0.40–0.59, ‘moderate’; and 0.60–1, ‘strong’. Statistical significance was assigned at *P* <0.05. R version 4.2.2 software (R Foundation, Vienna, Austria) was used for statistical analysis.

## RESULTS

### Analysis of DVH and plan parameters

In 17 out of 30 patients (56.7%), the dwell time weight of the needles to the tandem/ovoid was >20%. Needle-Lock plans were generated retrospectively for these 17 cases. Table 2 summarizes the DVH and plan parameters for all clinical and Needle-Lock plans with dwell time weights >20%. The CTV_HR_ volume was significantly smaller in the group with a needle dwell time weight of ≤20% than in the group with a needle dwell time weight of ≥20% (*P* < 0.01).

**Table 2:** Treatment characteristics for three groups

	(a) Clinical plan (DTW <20%) (*n* = 13)	(b) Clinical plan (DTW >20%) (*n* = 17)	(c) Needle-Lock plan (*N* = 17)	*P* value
				(a) vs (b)
CTV_HR_ volume (cc)	23.5 (10.8–42.1)	45.4 (14.8–163.3)		<0.01
Number of needles	1 (1–2)	3 (1–7)		
T Stage (8th edition of UICC/AJCC)				
T2a2	1	1		
T2b	12	9		
T3b	0	5		
T4a	0	2		
				(b) vs (c)
CTV_HR_ D_90_ (cGy)	887.0 (741.8–941.0)	829.3 (660.9–910.0)	829.3 (660.9–907.0)	0.50
CTV_HR_ V_100%_	100.0% (99.2–100.0%)	99.9% (97.0–100.0%)	99.5% (97.5–100.0%)	0.38
OARs D_2cc_ (cGy)				
Rectum	305.5 (101.1–438.6)	332.0 (168.0–537.5)	341.6 (176.0–721.7)	0.03
Bladder	516.6 (390.0–587.5)	536.0 (381.0–767.8)	580.0 (401.1–806.4)	0.02
Sigmoid colon	350.7 (150.7–465.3)	337.0 (117.9–584.3)	363.3 (112.7–593.4)	0.01
Small bowel	258.8 (0.0–440.1)	292.8 (81.1–518.0)	286.0 (88.5–588.0)	0.28
OI	0.332 (0.295–0.349)	0.341 (0.208–0.374)	0.346 (0.287–0.395)	<0.01
Needle weight	9.8% (2.8–20.0%)	61.3% (22.6–144.2%)	20.1% (19.7–20.4%)	<0.01

Values represent the median (range). Bold values indicate *P* value <0.05. UICC/AJCC = International Union against Cancer/American Joint Committee on Cancer, OI = overdose volume index, DTW = dwell time weight of needles to the tandem/ovoid.

A comparison of the Needle-Lock and clinical plans with needle dwell time weights >20% revealed that the Needle-Lock plans resulted in a significantly higher dose for the bladder, rectum and sigmoid colon D_2cc_. In the group with a needle dwell time weight of ≤20%, the median needle dwell time weight was 9.8%, but in the group with a needle dwell time weight of ≥20%, it reached 61.3%. Moreover, the Needle-Lock plans had significantly higher OI than the clinical plans.

**Fig. 4:**
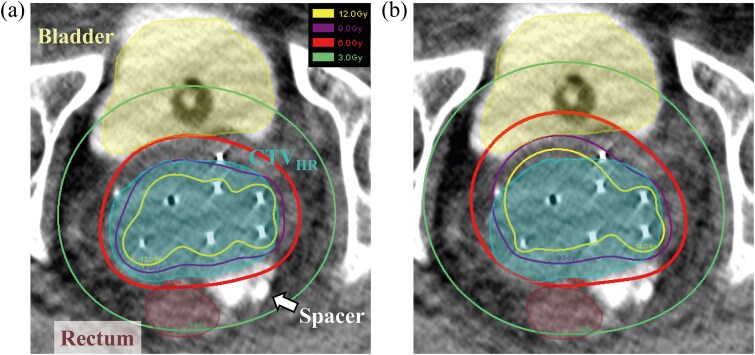
Dose distributions for patient 9. (a) Clinical and (b) Needle-Lock plans. The solid lines represent the isodose lines of 3, 6, 9 and 12 Gy. The arrow represents the hydrogel spacer.

Figure 4 shows the dose distributions of the clinical and Needle-Lock plans for patient 9 who had the largest CTV_HR_ volume (163.3 cc) with six needles inserted. The dose distribution of the clinical plan had a better fit with the CTV_HR_ than the Needle-Lock plan. The clinical plan was able to reduce the bladder dose. In the clinical plan, hyper-dose sleeve was within the CTV_HR_, while part of hyper-dose sleeve was located outside the CTV_HR_ in the Needle-Lock plan. Notably, the dwell time weight of the needles in the clinical plan for this patient was 81.2%, the highest among all patients. Conversely, the D_2cc_ of the bladder significantly increased by 37.8% in the Needle-Lock plan.

### Comparison of differences in the needle-lock plans with and without the hydrogel spacer using virtual OARs simulation

**Fig. 5:**
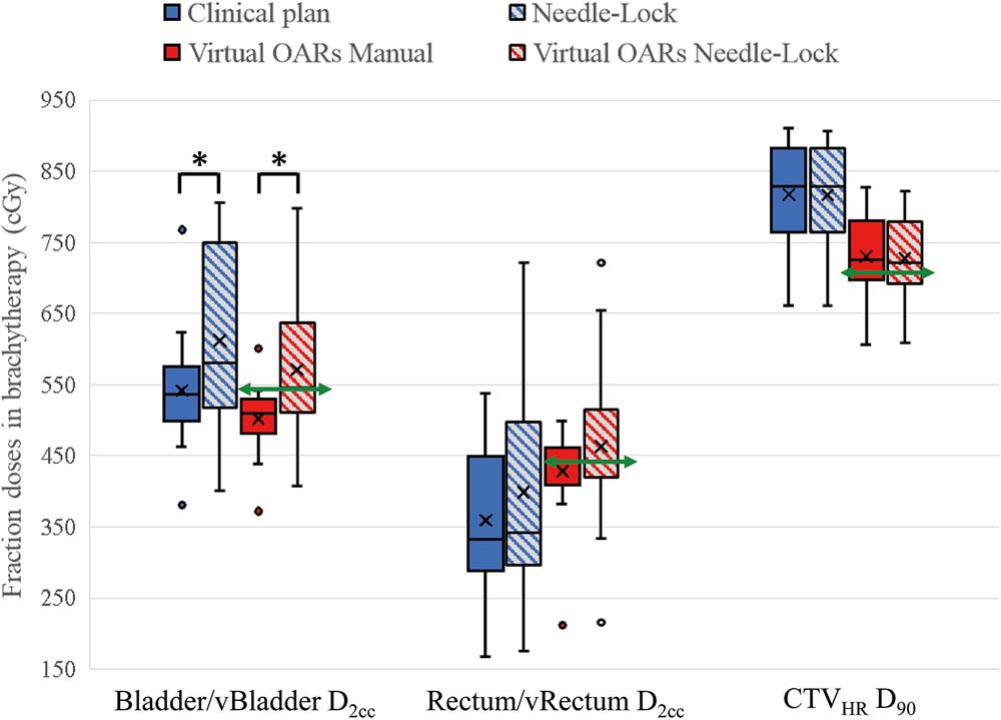
Box plots of the dose difference between the manual plans and the Needle-Lock plans for the OARs D_2cc_ and the CTV_HR_ D_90_. The bottom and top of the box indicate the 25th and 75th percentiles, respectively. The line inside the box represents the median, whereas the crossmark represents the average. The whiskers’ ends display the maximum and minimum values. The solid line represents the fractional dose constraints of each OARs only on the virtual OARs simulation. The asterisks indicate a statistically significant difference (*P* < 0.05).

Figure 5 shows box plots of the DVH parameters in the CTV_HR_ and OARs for manual and Needle-Lock plans. Regarding the fractional dose constraints in the virtual OARs simulation, shown by the green solid line in Fig. 5, violations of the constraints were observed in 6 cases for the vRectum, 1 case for the vBladder, and 7 cases for the CTV_HR_. Since the radiation oncologist determined whether to prioritize the dose constraints of the OARs or the CTV_HR_ for each case, there were cases where the constraints were not achieved. The details of the treatment parameters are listed in Table 3.

**Table 3:** Treatment characteristics in virtual OARs simulations

	Virtual OARs Manual	Virtual OARs Needle-Lock	*P* value
CTV_HR_ D_90_ (cGy)	726.0 (606.2–827.0)	721.0 (608.9–822.0)	0.68
CTV_HR_ V_100%_	98.6% (90.5–100.0%)	98.1% (90.7–99.7%)	**0.01**
OARs D_2cc_ (cGy)			
Rectum	442.0 (212.0–499.3)	442.0 (216.0–720.7)	0.12
Bladder	509.3 (371.5–601.1)	546.0 (408.1–798.4)	**<0.01**
Sigmoid colon	343.0 (98.0–501.3)	381.2 (91.0–556.4)	0.05
Small bowel	231.8 (70.0–508.2)	258.0 (73.0–517.2)	0.87
OI	0.319 (0.198–0.367)	0.331 (0.283–0.366)	**0.01**
Needle weight	52.9% (24.1–154.1%)	20.1% (19.5–20.4%)	**<0.01**

Values represent the median (range). Bold values indicate *P* value <0.05. OI = overdose volume index, DTW = dwell time weight of needles to the tandem/ovoid.

As with the use of the hydrogel spacer, when the needle dwell weight was limited to 20% in the virtual OARs simulation, the increase in D_2cc_ for the vBladder was observed with the statistical significance. When the hydrogel spacer was not used, the high-dose region occurred outside the CTV_HR_, so it could be seen that the dose constraints for the vRectum and the vBladder could not be met when the needle dwell time weight was limited. On the other hand, when the dose constraints for OARs were given priority by limiting the needle dwell time weight, it resulted in inadequate dose delivery to the CTV_HR_.

### Correlations between differences in each parameter and the number of needles

**Fig. 6:**
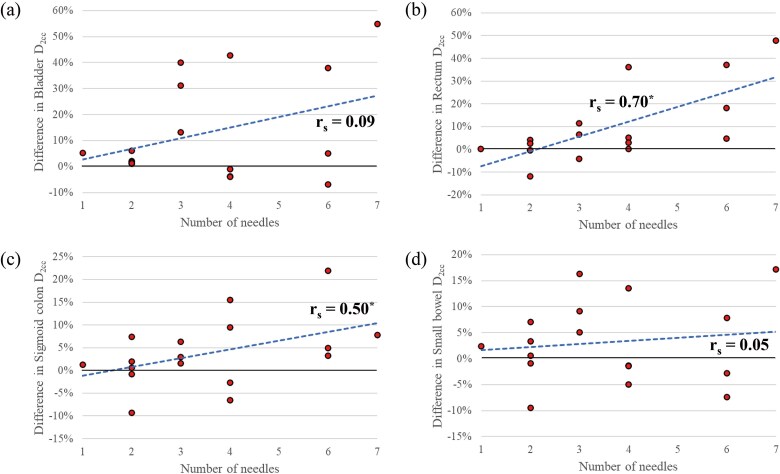
Correlation between the number of needles and differences in the clinical and the Needle-Lock plans for each OAR D_2cc_. (a) Bladder, (b) rectum, (c) sigmoid colon and (d) small bowel. The dashed line represents the linear regression line. The asterisks indicate a statistically significant difference (*P* < 0.05).

Figure 6 shows the differences in the D_2cc_ of the OARs between the clinical and Needle-Lock plans for the number of needles used in the HBT. For all OARs, the number of needles and variations in OAR D_2cc_ were positively correlated. Furthermore, Table 4 summarizes the results of the statistical correlation between differences in the clinical and the Needle-Lock plans for each OAR D_2cc_ using Spearman’s rank correlation coefficient. A strong correlation was found between the number of needles and the variation in the D_2cc_ of the rectum, and a moderate correlation was found between the variation in the D_2cc_ of the sigmoid colon.

**Table 4:** Correlation between differences in OARs D_2cc_ and the number of needles

	Spearman’s rank correlation coefficient (*r*_s_)	*P* value
Bladder	0.09	0.719
Rectum	0.70	<0.01^*^
Sigmoid colon	0.50	0.04^*^
Small bowel	0.05	0.85
vBladder	0.20	0.448
vRectum	0.63	<0.01^*^

A statistically significant difference is indicated by an asterisk (*P* value <0.05).

**Fig. 7:**
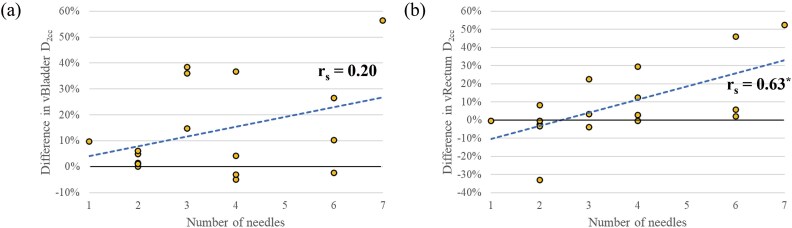
Correlation between the number of needles and differences in the manual and the Needle-Lock plans for each OAR D_2cc_ for the virtual OARs simulation. (a) vBladder, (b) vRectum. The dashed line represents the linear regression line. The asterisks indicate a statistically significant difference (*P* < 0.05).

Furthermore, the correlation analysis between the difference in the D_2cc_ of vRectum and vBladder with the limitation of needle dwell time weight and the number of needles was performed using Spearman’s rank correlation coefficient. Figure 7 shows the differences in the D_2cc_ of the virtual OARs between the virtual OARs Manual and the virtual OARs Needle-Lock plans for the number of needles. The statistically significant strong correlation was found between the number of needles and the difference in rectum and vRectum, regardless of whether the hydrogel spacer was used or not.

## DISCUSSION

In this study, we evaluated the impact of the needle dwell time weight constraint on the OAR dose and several HBT planning parameters.

The bladder, rectum and sigmoid colon D_2cc_ significantly increased when the dwell time weight of needles was limited to 20% (Table 2). Under the constraint of the needle dwell time, the tandem dwell time needs to increase to provide the prescribed dose to the CTV_HR_. Therefore, the dose distribution was further extended in the anterior–posterior direction than in the clinical plan, increasing the bladder and rectum dose. Although hydrogel spacers were injected, the rectal dose increased significantly when the needle dwell time weight was limited. As shown in Fig. 5, when there were no hydrogel spacers, the rectum dose was higher in the manual plans, and when the needle dwell time weight was limited, the rectum dose increased in some cases.

To avoid overdose outside the uterus, the latest practical guide limits the dwell time weight of needles to 10–20% [5]. However, the Needle-Lock plans resulted in a significant increase in OI compared to the clinical plans. This was because the prescription dose was mainly delivered to the CTV_HR_ by the tandem, whose dwell time weight constraints resulted in the incidence of overdose. Needle-Lock plans produced >200% of the overdoses occurring outside of the CTV_HR_ (Fig. 4). The clinical plans significantly reduced the hyper-dose sleeve volume using a median needle weight of 61.3% for the tandem/ovoid (Table 2). These results elucidated that the needle weight constraint can be potentially removed to reduce the incidence of overdoses and OAR doses. Compared with EBRT, HBT has less freedom in the shape of the dose distribution, and the clinical plan that satisfies the dose constraints was assumed to be the ideal shape of the dose distribution. Therefore, the method used in this study to perform inverse planning on the dummy isodose structure of the clinical plan used in this study while limiting the needle dwell time weight could be justified. To validate this approach in the future, it should be studied in more cases and at multiple institutions, but we think that this report should be important because it has raised awareness of the issue.

It is important to assess the cases in which the dwell time weight constraints of needles are not of concern. As shown in previous studies, the IGABT plan should be designed in a way to prevent overdoses outside the uterus [1, 2, 5, 9, 10]. Due to the needle weight constraint, the rectum doses increased as the number of needles increased (Figs 6 and 7). This correlation was also consistent with the additional virtual OAR analysis. These results clarified that when the needle dwell time is limited to 20%, the dose to the OAR increases consistently as the number of needles increases. In addition, for the bladder, which is close to the tandem, the dose increases of nearly 40% was observed even with three needles, regardless of the existence of the hydrogel spacer, due to the limitation of the needle dwell time weight. Based on the results of this study, we recommend the following procedure for optimizing the dose distribution. The initial generation of the dose distribution should be performed without any limitations on the needle dwell time, according to the institution’s protocol. Then, the radiation oncologist should evaluate where the hyper-dose sleeve regions are occurring by observing the dose distribution slice-by-slice and, if necessary, apply limitations on the needle dwell time weight. However, it should be noted that the optimal needle dwell time ratio can vary strongly depending on when brachytherapy is performed relative to EBRT, as tumor shrinkage in response to EBRT could affect the required needle dwell time ratio. In addition, the selection of applicator type, whether conventional tandem and ovoids or applicators dedicated to HBT, could affect the possible needle positions and number, which in turn would affect the optimal needle dwell time ratio. Therefore, the relationship between needle dwell time weight and dose distributions, which is the finding of this study, should be carefully considered in the context of specific treatment protocols, brachytherapy timing and applicator choices when applying it to any dose schedule or other brachytherapy applicators, such as Venezia, which were not used in this study. This study could not establish a correlation between the treatment outcomes and eliminating the constraint of needle dwell time weight. For this, we need to carefully monitor the treatment outcomes of the clinical plans to truly assess the appropriateness of eliminating the constraint of needle dwell time weight because allowing the needle dwell time weight escalation could potentially create larger high-dose volumes outside of the uterus, causing unexpected late radiation-related toxicities. However, the authors believe that if we design the dose distribution carefully so that the hyper-dose sleeve is only created inside of the tumor, it is clinically acceptable. Long-term treatment outcomes regarding the safety of this treatment approach will be reported in future studies. Another disadvantage of this study was the inadequate number of cases for each number of needles. It is therefore essential to increase the number of cases further and to investigate the correlation between the number of needles and the delivered dose to OARs associated with the constraint of needle dwell time weight. The conclusion of this study was strongly influenced by whether or not the needle insertion position was optimal. Our institution’s policy was to insert the needle in areas where the CTV_HR_ could not be adequately covered by the prescribed dose using intracavitary applicators alone. Prior to the first brachytherapy session, several radiation oncologists discuss and decide where to insert the needle based on the MR images from the MR scans taken immediately prior to the session and findings obtained from gynecological physical examinations.

The relationship between the constraint of the dwell time weight of the needle and OAR doses in HBT for uterine cervical cancer was investigated. Rectum doses increased as the number of needles increased, regardless of the existence of the hydrogel spacer. The constraint of needle’s dwell time weight resulted in increased overdoses >200% of the prescribed dose occurring outside the CTV_HR_ also increased. Therefore, this planning study demonstrated the superiority of eliminating the weight limit on the needle when necessary in order to ensure that bulky or irregularly shaped tumors are covered by adequate dose while maintaining the OAR doses within the dose constraints recommended by the guidelines.
